# SSU Ribosomal DNA-Based Monitoring of Nematode Assemblages Reveals Distinct Seasonal Fluctuations within Evolutionary Heterogeneous Feeding Guilds

**DOI:** 10.1371/journal.pone.0047555

**Published:** 2012-10-24

**Authors:** Mariëtte T. W. Vervoort, J. Arie Vonk, Paul J. W. Mooijman, Sven J. J. Van den Elsen, Hanny H. B. Van Megen, Peter Veenhuizen, Renske Landeweert, Jaap Bakker, Christian Mulder, Johannes Helder

**Affiliations:** 1 Laboratory of Nematology, Department of Plant Sciences, Wageningen University (WUR), Wageningen, The Netherlands; 2 Laboratory for Ecological Risk Assessment, National Institute for Public Health and the Environment (RIVM), Bilthoven, The Netherlands; 3 Laboratory for Soil and Crop Research (BLGG AgroXpertus), Wageningen, The Netherlands; American University in Cairo, Egypt

## Abstract

Soils are among the most complex, diverse and competitive habitats on Earth and soil biota are responsible for ecosystem services such as nutrient cycling, carbon sequestration and remediation of freshwater. The extreme biodiversity prohibits the making of a full inventory of soil life. Hence, an appropriate indicator group should be selected to determine the biological condition of soil systems. Due to their ubiquity and the diverse responses to abiotic and biotic changes, nematodes are suitable indicators for environmental monitoring. However, the time-consuming microscopic analysis of nematode communities has limited the scale at which this indicator group is used. In an attempt to circumvent this problem, a quantitative PCR-based tool for the detection of a consistent part of the soil nematofauna was developed based on a phylum-wide molecular framework consisting of 2,400 full-length SSU rDNA sequences. Taxon-specific primers were designed and tested for specificity. Furthermore, relationships were determined between the quantitative PCR output and numbers of target nematodes. As a first field test for this DNA sequence signature-based approach, seasonal fluctuations of nematode assemblages under open canopy (one field) and closed canopy (one forest) were monitored. Fifteen taxa from four feeding guilds (covering ∼ 65% of the free-living nematode biodiversity at higher taxonomical level) were detected at two trophic levels. These four feeding guilds are composed of taxa that developed independently by parallel evolution and we detected ecologically interpretable patterns for free-living nematodes belonging to the lower trophic level of soil food webs. Our results show temporal fluctuations, which can be even opposite within taxa belonging to the same guild. This research on nematode assemblages revealed ecological information about the soil food web that had been partly overlooked.

## Introduction

The biotic soil fraction is the source of major ecosystem services like water holding, nutrient cycling, and carbon sequestration [1]–[5] and is the bottom-up driving force of the ecosystem [6], [7]. Therefore, due to their differences in habitat-responses and multitrophic interactions [5]–[9], many terrestrial invertebrates are valuable ecological indicators [10]. However, irrespectively of the environmental characteristics we wish to highlight, the high biodiversity in soils and sediments [11], [12] forces us to choose a subset that is representative for biological soil quality. Ecological criteria to select indicator groups should include a) distribution across multiple trophic levels, b) methodological interpretability of qualitative and/or quantitative changes, and c) ease of sampling standardization. Soil nematodes meet these criteria.

These vermiform invertebrates, mostly with body lengths ranging between 0.2 and 2.5 mm [13], are present in densities up to several millions individuals per square meter, and are easily extractable from the topsoil. Their trophic diversity encompasses all the three energy channels distinguishable within the soil food web: the plant-feeding, the bacterial-feeding, and the fungal-feeding pathway (*e.g*. [14]). Because of their highly interconnected positions in the detrital soil food web, nematode communities reflect microbial resources, especially the bacterial and the fungal communities [5], [15], [16], soil fertility and management [14], [17], [18]. Simple food webs with few trophic levels, as those in our study, show more specialization and less omnivory because occurring species (here, nematodes) have a much higher probability of consuming at one single trophic level [19], [20]. Hence, nematodes are a natural avenue to examine the spatial and temporal variance of such food web configurations.

Moreover, the nature and rate by which nematodes respond to changes in the (a)biotical soil condition varies amongst different families and genera. At community level, this variation in responsiveness reflects itself changes in the numerical abundance, species composition, feeding traits and trophic distribution. The interconnected positions in the soil food web, in combination with taxon-specific responsiveness towards environmental stressors, make these invertebrates suitable as indicators.

Over the last decade, substantial progress has been made in collecting phylum-wide genetic information of nematodes [21]–[23]. This resulted in a small subunit ribosomal DNA-based (SSU-rDNA) framework covering a substantial part of the biodiversity for terrestrial nematode communities in temperate climate zones. Other studies independently introduced molecular tools to analyze nematode communities using qualitative or semi-quantitative techniques such as direct sequencing [24], PCR DGGE [25], and T-RFLP [26].

For DNA-based quantitative community analysis, the effect of the (unknown) life-stage distribution within individual taxa should be considered. In the past, nematodes were thought to exhibit cell constancy; all individuals of a given species have the same number of cells. However, at least for one organ, the epidermis, this was shown to be incorrect [27]. During their development from first or second juvenile stage to adult stage, the number of somatic cells appears to show less than a two-fold increase. In case of *Caenorhabditis elegans* (Rhabditidae) the number of non-gonadal cells increase from ≈ 550 (first stage juveniles) to ≈ 810 (mature hermaphrodite) [28], while *Panagrellus silusiae* (Panagrolaimidae) the number of somatic nuclei was shown to increase from ≈ 410 (second stage juvenile) to ≈ 590 (adult) [29]. Although data on this issue are scarce (but see [30] for cryptic Rhabditidae), we hypothesize here that it is possible to relate quantitative PCR data to the number of individuals of a given family at a logarithmic scale without knowing their exact distribution over the life stages. If this is true, a SSU-rDNA quantitative community analysis will define emergent characteristics of the nematofauna and can provide one common currency to assess the soil food web by comparing the quantitative PCR output of differently-sized nematodes.

According to Neher [31], more research is needed on nematodes in natural and agricultural soils to test ecological hypotheses. Hence, we chose two adjacent ecosystems to investigate the extent to which the SSU-rDNA tool allows monitoring of soil nematode assemblages in the South of the Veluwe region (central Netherlands). In such a way, we were able to establish the degree of convergence of the soil ecological condition 30 years after ending the agricultural practices by a direct comparison between the compositions of the nematode community in the previously disturbed area (‘former arable field’) and the adjacent undisturbed control (‘mature beech forest’), as recommended in [31]. During 10 months, nematodes were sampled 18 times with intervals of 2–4 weeks. 15 families or genera were detected (monophyletic groups in a phylum-wide SSU rDNA framework that includes ≈ 2,400 taxa as described in [23]); within them most feeding guilds were represented. Occurrence of specialized nematodes parasitizing vascular plants greatly depends on the structure of the rhizosphere, hence on the composition of the vegetation and as for the Maturity Index [32] they were not taken into consideration in this nematological research. Shortly, recurring DNA patterns (motifs) were identified for family or genus-specific groups and PCR primers with identical annealing temperatures were developed. We show that nematode assemblages can be monitored frequently using standard molecular laboratory equipment and that this method has the potential to contribute to the full exploitation of this abundant and diverse group of metazoans.

## Materials and Methods

### Study Area

Seasonal fluctuations of non-parasitic nematode assemblages were studied in De Planken Wambuis, a nature reserve located on the Veluwe, the largest moraine complex in The Netherlands. Due to the absence of endangered and/or protected species, this investigated area of approximately 100 m length is not protected by law and no specific permits were required. Sandy soil samples were taken from two sites: a 30-year-ago abandoned arable field, known as Dennenkamp (52° 03′ N, 5° 80′ E), and an adjacent more than 100-year-old *Fagus sylvatica* forest.

The former arable field (sampling area 2.5 ha; further referred to as ‘field’) is a relatively open area with a *Plantagini-Festucion* association (*sensu* [33]) growing on a soil with pH of 5.7; more characteristics of this site have been published in Holtkamp *et al*. [34]. The pristine beech forest (further referred to as ‘forest’) with typical medium humified humus, hereafter moder soil (pH≈3.7), is characterized by a scarce understory (sampling area 1.5 ha). The precipitation and temperature data were registered by a weather station by the Royal Dutch Meteorological Institute (KNMI Station 06275, 45 m a.s.l., about 7 km from Dennenkamp).

### Sampling and Nematode Extraction

Nematode assemblages were monitored throughout 2009 in an abandoned field and an adjacent pristine beech forest. On these sites, the upper 25 cm of the soil were sampled 18 times from March 17 (week 1) until December 18 (week 40). The humus fraction was still observable as a stratified layer in the forest moder (partly decayed, to some extent mixed with the mineral horizon). At eighteen time points (every 2–4 weeks), we randomly took four composite soil samples from the field, and two composite samples from the adjacent forest. Each sample consisted of 8–10 cores (Ø 1.5 cm, depth 25 cm) taken from a surface of ∼0.25 m^2^ and thoroughly mixed. Nematodes were extracted from 100 ml of soil using an elutriator [35]. Nematode density was estimated by counting two subsamples per sample at low magnification (classical analysis); after counting, these subsamples were poured back into the original suspension.

### Selection of Nematode Taxa

To make a selection of monitored taxa for this study, suspensions from both sites were analysed microscopically twice (week 1 and week 39, 2009). In total, 38 genera *sensu* Bongers [36] were identified in the field and 25 in the forest (Table 1). Fifteen nematode taxa (families and genera; Table 2) were selected based on molecular resolution, trophic ecologies, and sensitivities towards environmental disturbances. This taxonomic selection at genus level covers 59% of the field and 72% of the forest nematode biodiversity (excluding the obligate plant parasitic genera; Table 1).

**Table 1:** Overview of nematode diversity at genus level (microscopic analysis) in the topsoil (depth 0–25 cm) of the former arable field and the adjacent pristine beech forest.

Genus	Family	qPCR analysis	Range	Field	Forest
*Achromadora*	Achromadoridae			+	
*Acrobeles*	Cephalobidae	q	r	+	
*Acrobeloides*	Cephalobidae	q		+	+
[*Aglenchus*]	[Tylenchidae]				+
*Alaimus*	Alaimidae	q	r	+	+
*Anaplectus*	Plectidae	q	r	+	
*Aphelenchoides*	Aphelenchoididae	q	r	+	+
*Aphelenchus*	Aphelenchidae	q	r	+	
*Aporcelaimellus*	Aporcelaimidae			+	
*Bunonema*	Bunonematidae				+
[*Cephalenchus*]	[Tylodoridae]				+
*Cephalobus*	Cephalobidae	q		+	
*Cervidellus*	Cephalobidae	q	r	+	+
*Clarkus*	Mononchidae	q	r	+	
*Coomansus*	Mononchidae	q		+	
[*Coslenchus*]	[Tylenchidae]				+
*Cylindrolaimus*	Diplopeltidae			+	
*Diphtherophora*	Diphtherophoridae	q	r	+	
[*Ditylenchus*]	[Anguinidae]			+	+
*Eucephalobus*	Cephalobidae	q	r	+	+
*Eudorylaimus*	Qudsianematidae				+
*Eumonhystera*	Monhysteridae	q	r	+	+
[*Filenchus*]	[Tylenchidae]			+	+
*Geomonhystera*	Monhysteridae			+	
[*Helicotylenchus*]	[Hoplolaimidae]			+	
[*Malenchus*]	[Tylenchidae]				+
[*Meloidogyne*]	[Meloidogynidae]			+	
*Mesorhabditis*	Mesorhabditidae			+	
*Metateratocephalus*	Metateratocephalidae	q	r	+	+
*Microdorylaimus*	Qudsianematidae			+	
*Nygolaimus*	Nygolaimidae			+	
*Panagrolaimus*	Panagrolaimidae			+	
*Plectus*	Plectidae	q	r	+	+
[*Pratylenchus*]	[Pratylenchidae]			+	
*Prismatolaimus*	Prismatolaimidae	q	r	+	+
*Pungentus*	Nordiidae			+	
*Rhabditis*	Rhabditidae			+	+
*Steinernema*	Steinernematidae				+
*Teratocephalus*	Teratocephalidae	q	r	+	+
*Thonus*	Dorylaimidae	q	r	+	+
*Tylencholaimus*	Tylencholaimidae			+	+
[*Tylenchorhynchus*]	[Belonolaimidae]			+	
[*Tylenchus*]	[Tylenchidae]			+	+
*Tylolaimophorus*	Diphtherophoridae	q	r	+	+
*Wilsonema*	Plectidae	q		+	+
**Total # of genera**	**45**	**20**	**16**	**38**	**25**

Obligate plant-parasitic nematodes are shown in grey [taxa ara given in brackets] and are not included in the molecular part of this research. Only the genera marked by ‘q’ are included in the quantitative PCR analysis and for most of these genera quantitative ranges (‘r’) are available (see Fig. 4 and text for more details). For the taxonomy of the families we adhered to De Ley *et al*. [66].

### DNA Extraction and Purification

Nematode suspensions (100 ml) were concentrated by centrifugation at 4,000 rpm, supernatant was removed until an end-volume of approximately 1.5 ml. This volume was further concentrated in a small vial at 14,000 rpm. The supernatant was removed until the final volume of 140 µl was reached. Subsequently, like in Holterman *et al*. [22], an equal volume of nematode lysis buffer was added. As an internal standard, 20 µl of mammalian DNA (20 ng/µl) was included. Lysis took place in an oven at 65°C for two hours. Lysates were purified using a glass fiber-based DNA extraction procedure (essentially according to Ivanova *et al*. [37]). Purified nematode community DNA was eluted from the filter with T_10_E_1_ (10∶1, 1 M Tris and 0.5 M EDTA) and immediately used or stored at −20°C. These purified lysates were used for quantitative PCR analysis. We kept this DNA extraction procedure consistent for all samples in our study to ensure full comparability of results ([38], note to their S3).

### Design and Testing of Family- and Genus-specific Primers

For the development of taxon specific PCR primers, a molecular framework consisting of ≈ 2,400 (nearly) full-length SSU rDNA sequences representing all major groups of terrestrial nematodes was used. ARB, a LINUX-based software package [39], was used to design family and/or genus-specific primers. Most nematode families appeared as monophyletic groups in a SSU rDNA based phylogenetic tree [23], and PCR primers were developed on the basis of taxon-specific motifs. In contrast, for some polyphyletic taxa – *e.g.*, fungivorous Diphtherophoridae – separate specific primer combinations were developed for each of the constituting genera. In the case of the poly- and paraphyletic Rhabditidae, embracing 27 genera according to the Fauna Europaea [http://www.faunaeur.org (Accessed 2012 June 6)], no comprehensive DNA barcodes could be generated at such a large family level, albeit for some monophyletic genera, specific primers can still be developed.

When designing the primer combinations, the annealing temperature of the oligonucleotides was assessed *in silico* using the program MELTING [40]. For each nematode taxon (family or genus), the specificity of multiple (up to 5) primer combinations was checked with recombinant SSU rDNA fragments from target(s) and close non-target(s) as identified by ARB (details in [41], [42]). Apart from the specificity requirements, primer combinations were designed to have an optimal annealing temperature (T_a_) of 63°C. Based on an experimental temperature range test, only target-specific primer combinations with a sharp optimum were selected. This approach allows for a quantitative detection of combinations of taxa with the same PCR temperature profile.

Primer combinations were tested in 25 µl containing 3 µl of 1,000 times diluted template (final concentration: 10 ng/µl), 1 µl of each of the taxon-specific primers (final concentration for each primer: 200 µg/µl), 7.5 µl Milli-Q water and 12.5 µl Absolute SYBR Green Fluorescein Mix (Thermo Fisher). For amplification on a thermal cycler (Bio-Rad iQ5), the following quantitative PCR temperature profile was used: 95°C, 15 min followed by 60× (95°C, 30 sec; 63°C, 1 min; 72°C, 30 sec) followed by a melting curve program 47× (15 sec from 72 to 95°C with steps of 0.5°C).

For each taxon, one primer combination was selected on the basis of optimal specificity (*i.e.*, largest ΔC_t_) between target(s) and close non-target(s); assays with a ΔC_t_ lower than 12 were discarded. Here, C_t_ value is defined as the number of PCR cycles (‘C’) at which the reporter dye emission intensity exceeds a predetermined threshold (‘t’). In case of similar specificities, primer combinations with lowest C_t_ value per unit of template were preferred.

### Quantitative PCR on Total Nematode Community DNA

Each purified lysate (DNA extract from 100 ml elutriated soil) was used as template with 15 primer combinations on a thermal cycler (Bio-Rad iQ5). A separate primer combination was used to quantify the internal standard (mammalian DNA) in each sample to estimate the efficiency of the lysis and purification procedure. Reaction volume of the quantitative PCR was 25 µl containing 3 µl of 50 times diluted template, 2 µl taxon-specific primers (end concentrations 200 µg/µl), 4.5 µl PVP40, 12.5 µl Absolute SYBR Green Fluorescein Mix (Thermo Fisher). The following quantitative PCR protocol was used: 95°C, 1 min followed (as before) by 60× (95°C, 30 sec; 63°C, 1 min; 72°C, 30 sec) followed by a melting curve program 47× (15 sec from 72 to 95°C with steps of 0.5°C).

### Relationships between C_t_ Values and Numbers of Target Nematodes

The quantitative PCR output is expressed in C_t_ units. The copy number and quantities of the target template are inversely proportional to C_t_ and can be calculated by direct comparison with C_t_ values for known standards [43], [44]. In order to get these standards, quantitative series of microscopically identified nematodes (mostly to genus level) were sampled. Vials containing 25 µl sterile water with 1, 5, 10, 50, or 100 hand-picked nematodes were supplemented with an equal volume of lysis buffer (0.2 M NaCl, 0.2 M Tris-HCl [pH 8.0], 1% (v/v) β-mercaptoethanol) and 800 µg/ml proteinase-K. Lysis took place in a Thermomixer (Eppendorf) at 65°C as described by Holterman *et al*. [22]. Quantitative PCR reactions were performed as described above: 3 µl of 1000x diluted lysate, 1 µl of each taxon-specific primer (end concentrations of both primers 200 µg/µl), 7.5 µl Milli-Q water, and 12.5 µl Absolute SYBR Green Fluorescein Mix (Thermo Fisher). In the case of family-specific primers, calibration curves were generated for the major genera within each family.

### Data Analysis

The total numbers of nematodes were log transformed and an overall comparison (including all sampling times) was made between the two ecosystem types (‘forest’ and ‘field’) using *t*-test (equal variances not assumed, α = 0.05). To visualize seasonal patterns and site-dependent differences, trend lines are shown for each family or genus per location. Inter-site comparisons were made for each of the detected families and genera and for the two basal trophic guilds (here as summed ‘bacterivores’ and summed ‘fungivores’) using independent Mann Whitney-U test (α = 0.05). To get the temporal variation of the nematode community between our two habitats, a partial Mantel analysis was performed.

## Results

### Seasonal Dynamics and Site-specific Differences in Nematode Communities

**Figure 1:**
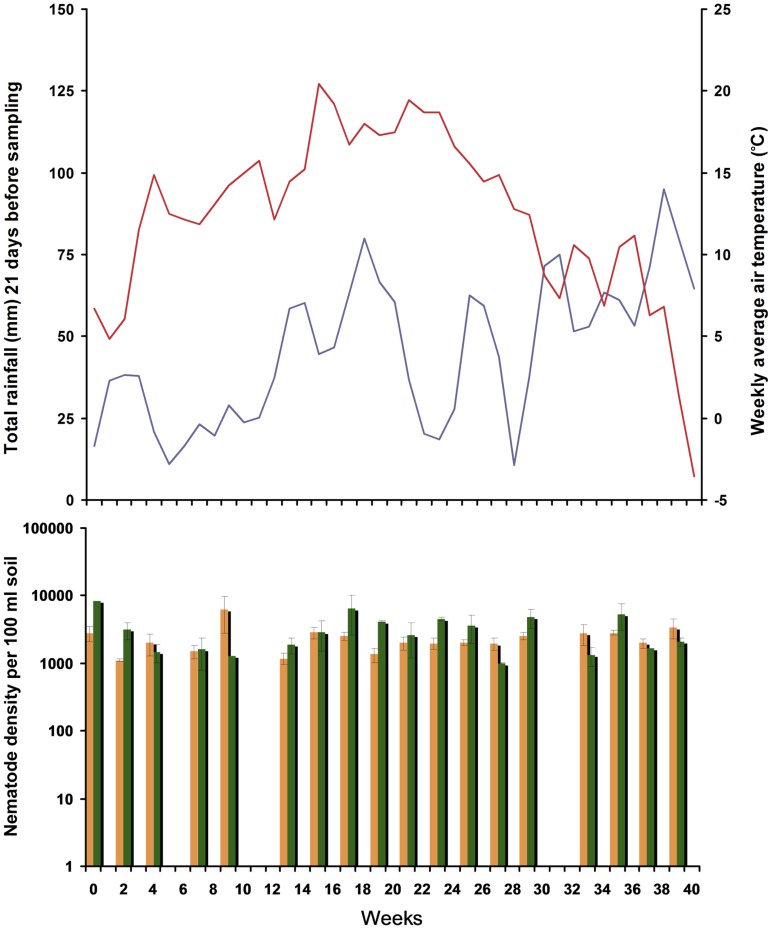
Precipitation and temperature in relation to total nematode densities in open (field) and closed (forest) canopies. Weekly averages of daily temperature (red) and total rainfall over 21 days before sampling (blue) as measured by the Royal Dutch Meteorological Institute (KNMI) are shown above. At the bottom, average nematode densities per 100 ml of soil from a since 25 years abandoned arable field (open canopy, yellow bars) and adjacent pristine beech forest (close canopy, green bars) are given. Sites sampled in 2009 at regular intervals between March 17 (week 1) and December 18 (week 39).

While the air temperature fluctuated between 20.4°C (week 14) and −3.5°C (week 39) and the cumulative rainfall of the latest 21 days before sampling (*sensu* [45]) fluctuated between 11 and 95 mm (Fig. 1, upper panel), variation of the total nematode density (Fig. 1, bottom panel) was rather low (Coefficient of Variation equals 48.1% in the field and 63.1% in the forest). The field had a lower density of nematodes in comparison to the forest (averages per 100 ml elutriated soil were 2,392±1,151 SD *versus* 3,222±2,033 SD individuals; unweighted *t*-test *P* = 0.023).

Composition of the soil nematode assemblages was determined microscopically from two composite suspensions (Table 1). Among these genera and families, taxa that appeared as monophyletic groups in a SSU rDNA-based molecular framework [23] were chosen for further investigation. From the basal level of the soil food web, 12 taxa were selected to be addressed in the next part, namely 8 bacterivores (7 families and 1 genus) and 4 fungivores (2 families and 2 genera), and from the trophically higher level, 3 taxa were selected, *i.e.* 2 predatory families (here: Mylonchulidae and Mononchidae M3) and one omnivore family (here: Dorylaimidae D3), for monitoring using real time PCR (Table 2). Primary data about densities of individual taxa at each of the time points (average and standard error) are given in the Table S1.

**Table 2:** Molecular overview of the nematode families and genera monitored in our study.

Nematode family/genus	ΔC_t_	Relationship between C_t_ value andlog (# target nematodes):			# gener^*^
		C_t_ = a ×log_10_ [# nematodes] + b			
		a	b	*R* ^2^	
**Alaimidae (B)**	N/A	−3.31	25.47	0.996	1
**Aphelenchidae (F, FP)**	42	−4.31	17.53	0.995	1
**Aphelenchoididae (F, FP )**	20	−3.06	24.09	0.992	1
**Cephalobidae (B)**	17	−4.21	21.95	0.855	3
**Diphtherophoridae (F) :**					
* Diphtherophora*	18	−3.22	19.18	0.926	
* Tylolaimophorus*	N/A	−3.02	22.36	0.984	
**Dorylaimidae (O)**	N/A	−5.90	17.30	0.859	1
**Metateratocephalidae (B)**	26	−5.09	24.40	0.954	2
**Monhysteridae (B)**	23	−4.25	21.06	0.954	1
**Mononchidae (P)**	18	−2.94	15.19	0.990	1
Mylonchulidae (P) **	N/A	−4.02	12.03	0.977	1
**Plectidae (B) :**					
Plectidae excl. *Anaplectus*	34	−1.93	26.82	0.989	1
* Anaplectus*	27	−3.33	21.03	0.949	
**Prismatolaimidae (B)**	13	−5.13	21.64	0.999	1
**Teratocephalidae (B)**	N/A	−4.41	25.13	0.999	1

Specificity of primer combinations is expressed as the gap between the C_t_ value of the latest target and the C_t_ value of the earliest non-target (ΔC_t_ expressed in number of PCR cycles). For relationship between C_t_ value and number of target nematodes see Fig. 4, and Materials and Methods in ‘*Relationships between C_t_ values and numbers of target nematodes*’.

B: bacterivore, F: fungivore, FP: facultative plant parasite (only for nematodes where this guild occurred in combination with fungivory), O: omnivore, P: predator; N/A: no quantitative PCR signal produced by non-target(s); ^*^: number of genera within one family assessed by qPCR (families as in De Ley *et al*. [66]); **: as *Mylonchulus* is expected to occur in this area, its family has been included as additional taxon.

**Figure 2:**
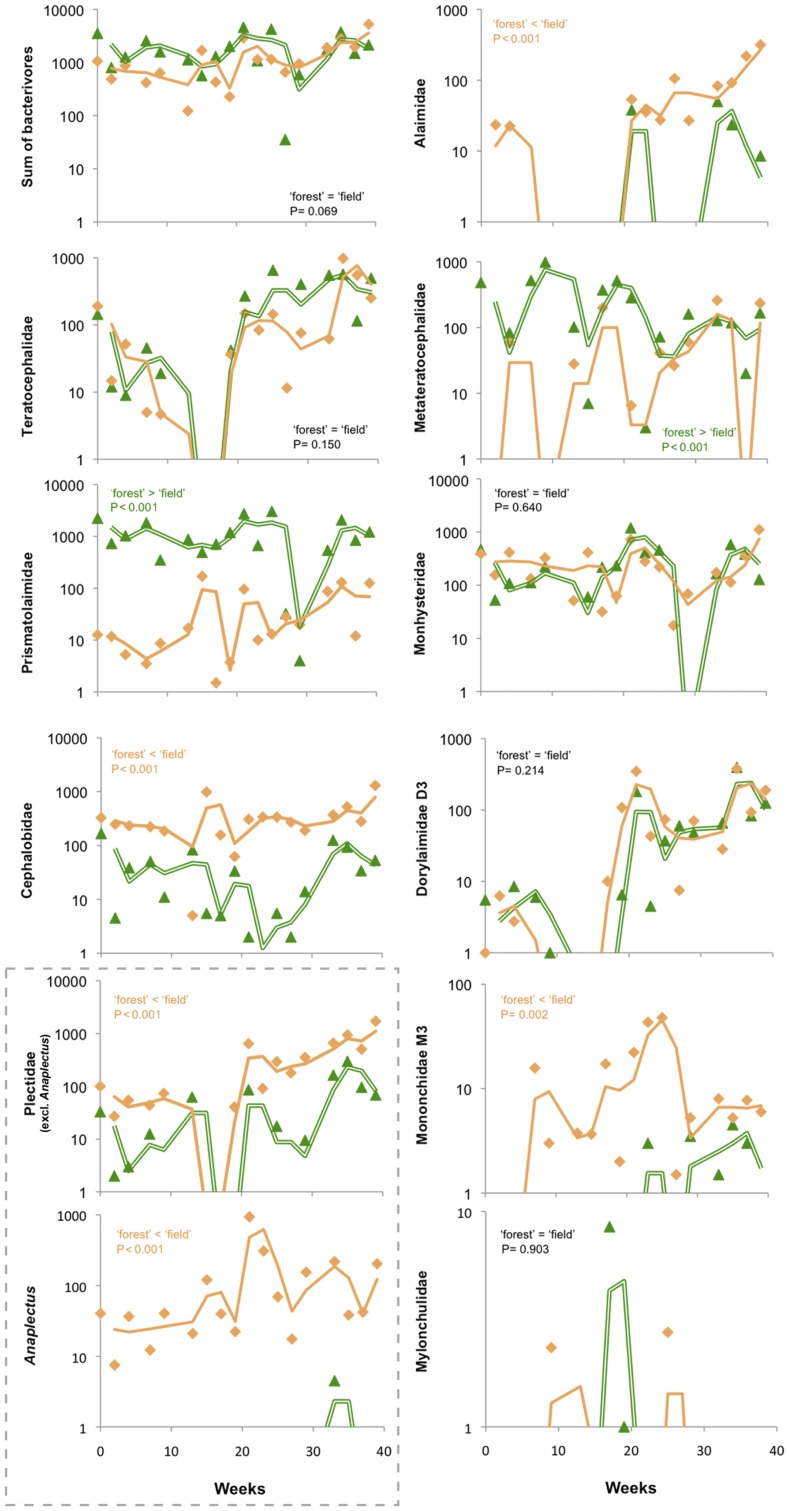
Temporal patterns of bacterivorous, omnivorous and predatory nematode families. We determined DNA-based variation in the nematode densities per 100 ml soil (note differences in *y*-axes) of representatives from seven bacterivorous families: Teratocephalidae, Prismatolaimidae, Cephalobidae, Plectidae (*i.e.*, all Plectidae excl. *Anaplectus* and ‘*Anaplectus*’, both in a dashed gray box), Alaimidae, Metateratocephalidae, Monhysteridae; the omnivorous family Dorylaimidae (D3 region *sensu* [65]); and the predatory families Mononchidae (M3, [65]) and Mylonchulidae. Sampling weeks as *x*-axes (constant scales); samples from the field are represented by orange triangles and samples from the forest by green diamonds. Trends are given as two-period moving averages: the averaged 2^nd^ and 3^rd^ data points are portrayed by the 1^st^ data point and so forth.

In contrast to the total nematode densities, individual taxa show distinct temporal and site-specific patterns. The seasonal fluctuations for 7 bacterivorous families are shown in Fig. 2 (*colonizer-persister cp* ranking as in [32], [46]): Teratocephalidae (*cp-*3), Prismatolaimidae (*cp-*3), Cephalobidae (*cp*-2), all in the left panel; Plectidae (*cp-*2) and the genus *Anaplectus* (*cp*-2), both in the red box; Alaimidae (*cp*-4), Metateratocephalidae (*cp*-3), and Monhysteridae (*cp-*2), all in the right panel. In particular, bacterivores show distinct temporal patterns in abundances in the two habitats, but also a taxon dependency was observed (Fig. 2). For instance, comparable trends are detectable for all Teratocephalidae (*i.e*., *Teratocephalus*, being Teratocephalidae monogenic).

Over the entire season, no significant differences between the two habitats were observable in the case of *Teratocephalus* and Monhysteridae (α = 0.05). Two families, Prismatolaimidae and Metateratocephalidae, were consistently more abundant in the forest, whereas Alaimidae, Cephalobidae, members of Plectidae and the genus *Anaplectus* were present in significantly higher densities in the field (Fig. 2). However, if the densities of these bacterivorous taxa are taken together into a single feeding guild (*Ba_x_*, bacterivores with *cp* value *x sensu* Ferris *et al*. [14]) no significant difference was detectable between the two sites (α = 0.05), despite the remarkable functional differences within bacterial-feeding nematodes known from literature [4], [9].

**Figure 3:**
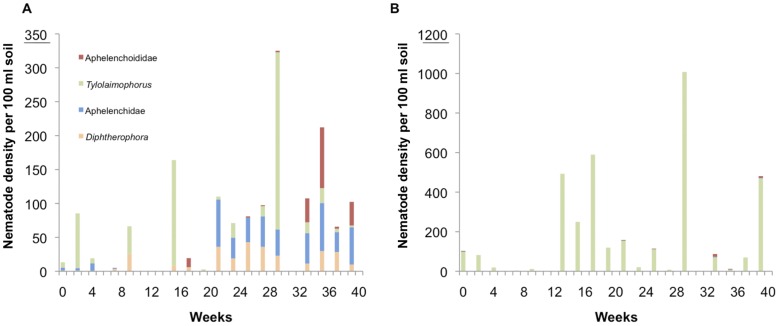
Temporal patterns of fungivorous nematode families. Seasonal variation in densities for fungivores in the field (**A**) and in the forest (**B**). Please note that the *y*-axis scales differ. Aphelenchidae (Aphe, blue), Aphelenchoididae (Acho, red), and two genera belonging to Diphtherophoridae –*viz*. *Tylolaimophorus* (Tylo, green) and *Diphtherophora* (Diph, yellow)– show different patterns over the seasons between open and close canopies. As these taxa represent all observed fungivores, a partial Mantel analysis performed in a matrix describing the community structure in the field (open canopies, matrix Y) and in the forest (close canopies, matrix X) using the squared Euclidean distance was performed using the total entries and the same set of entities. A positive association between the matrices is indicated over the seasons by observed Z greater than average Z from randomized runs (*P* = 0.0297).

In parallel, three fungivorous families were monitored as well; Aphelenchidae (*cp*-2), Aphelenchoididae (*cp*-2), and Diphtherophoridae (*cp*-3). Ribosomal DNA sequences suggest that Diphtherophoridae are not monophyletic [23]. Hence, representatives of the two constituting genera, *Tylolaimophorus* and *Diphtherophora*, were detected separately. The composition of this guild is site-specific: whereas the forest was dominated by *Tylolaimophorus,* the fungal pathway of the nematofauna in the field was more diverse (Fig. 3), although *Tylolaimophorus* remained predominant. Their densities showed strong temporal fluctuations: from week 20 onwards, all these fungivorous families were present in the field, although at low levels.

### SSU rDNA-based Assays for the Detection of Nematode Taxa – qualitative Aspects

**Figure 4:**
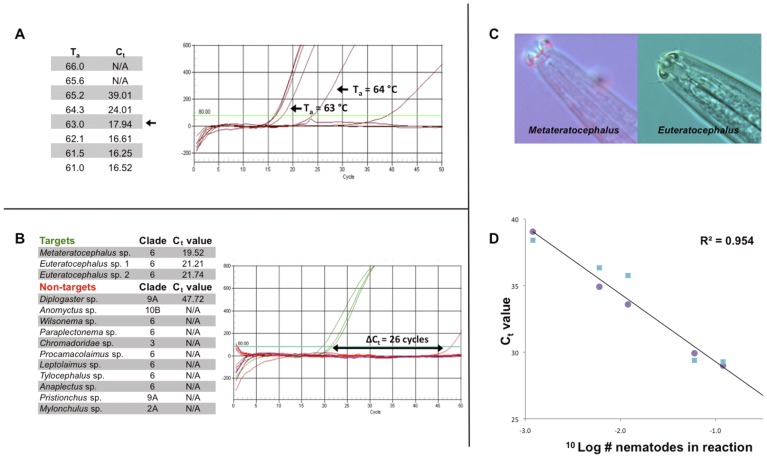
Development and testing of a nematode family-specific primer combination. Here we use the Metateratocephalidae (one bacterivorous family harboring the *Metateratocephalus* and *Euteratocephalus* genera) as an example of primer development. (**A**) All primers were designed to have optimal annealing temperature (T_a_) of 63°C, with C_t_ values varying at temperatures above and below the target T_a_. (**B**) Specificity test of a Metateratocephalidae primer combination with plasmid DNAs from three target species, SSU rDNA fragments from 11 potential false positives (as selected by ARB [39]) and a negative water control. Clade numbers are according to Van Megen *et al*. [23]. In the quantitative PCR graph the gap between the target and the non-target signal (ΔC_t_) is shown. (**C**) Pictures of the head region of a representative of both genera. (**D**) The relationship between C_t_ values and numbers of nematodes for quantification of densities. A linear relationship between C_t_ values and numbers of nematodes till ^1^/_1,000_ part of a single nematode is shown (equivalent to a single nematode cell harboring ∼50 copies of the ribosomal DNA cistron). Hand-picked individuals of *Metateratocephalus* (purple circles) and *Euteratocephalus* (blue squares) were used to quantify the Metateratocephalidae-specific primers.

Soil samples typically contain 30–60 nematodes species, and the composition of nematode assemblages is highly dependent on soil conditions [13], [47]. Keeping this degree of complexity in mind, the development of such a molecular community analysis tool requires a comprehensive SSU rDNA database [23]. A selection of 15 taxa was made with representatives of four major guilds: i– bacterivores, ii– fungivores, iii– omnivores, and iv– carnivores. The strategy followed for the development of specific PCR primers is exemplified here by the Metateratocephalidae, a bacterivorous family harboring two genera, *Metateratocephalus* and *Euteratocephalus* (Fig. 4). SSU rDNA sequence motifs were used to design primers with an annealing temperature (T_a_) of 63°C. To optimize the foreseeable specificity, selected primer combinations showed a sharp increase in C_t_ (threshold cycle) upon further T_a_ increase (Fig. 4A). ARB software [39] was employed to identify potential false positives, and plasmids harboring relevant SSU rDNA fragments were used for testing PCR primer combinations. Taxonomically, it must be mentioned that potential false positives are not *per se* related to targets, underlining the plea for a phylum-wide database. In case of the most optimal Metateratocephalidae primer combination, the smallest gap between the target and the non-target smallest (ΔC_t_) measured 26 cycles (Fig. 4B). This value was determined for all primer combinations (Table 2).

### SSU rDNA-based Assays for the Detection of Nematode Taxa – Quantitative Aspects

To establish the relationship between a C_t_ value (the primary output of a quantitative PCR reaction) and the corresponding number of target nematodes (here, members of the Metateratocephalidae), two series of handpicked individuals were generated. The resulting dataset, five C_t_ values for each *Metateratocephalus* and *Euteratocephalus* (Fig. 4C), was used to define the slope and the *y*-intercept of the regression line describing the linear relationship between log (# nematodes) and the corresponding C_t_ values (Fig. 4D). As an assessment of the goodness of fit, *R* ^2^ values are given for each taxon. Although families may harbor more genera than the number given in Table 2, the values presented here only aim to indicate the number of genera that were observed at this particular study area. Considering the life-stage distribution for each taxon (with differences in DNA contents for individual life stages), a taxon-specific degree of uncertainty regarding the exact densities might occur. However, seen the *R* ^2^ values for each taxon (Table 2), we might assume that the SSU rDNA-based densities reflect the actual densities assessed by classical nematological analysis.

### Quantitative Coverage of Environmental Samples by a 15-taxa Nematological Analysis

**Figure 5:**
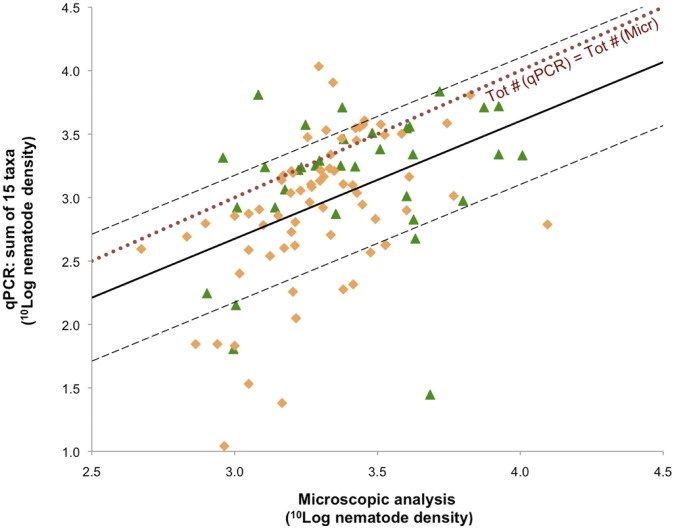
Quantitative coverage of the DNA-based tool using environmental samples. Logarithm of the total of individuals as detected by optical microscopy (*x*-axis) plotted against the logarithm of the total of individuals as estimated by quantitative PCR (*y*-axis). The correlations of quantitative PCR with classical analyses seem to be accurate, with no Studentized residuals higher than | 2 |. The solid line shows the trend of all data and the two dashed lines show the boundaries of one-order-of-magnitude precision. The dotted line represents an equal amount of nematodes for both methods. Such a coverage is expected to be lower than 100% as obligate plant parasites were not included, although the fungivorous Aphelenchidae and Aphelenchoididae may harbor facultative plant parasites as shown in Table 2. Given that taxa like Rhabditidae, Qudsianematidae or Nordiidae appear to be both poly- and paraphyletic [23], [65], no rDNA-based detection assay on family level could be developed for those nematodes.

If all taxa present in the samples were covered by this novel quantitative PCR-based community analysis tool, the sum of the densities should equal the total nematode numbers as given in Fig. 1. To check the quantitative coverage of the 15-taxon analysis tool, the total number of nematodes as determined microscopically was compared with the total numbers as estimated by quantitative PCR. We constructed a red dotted line to show virtual data at which the total number of nematodes is equal irrespective whether determined by microscopy or by quantitative PCR (Fig. 5) and one solid line to connect all the points. Ideally, a dataset should not exceed 0.5 log value from the latter solid line, allowing a precision of ±0.5 order of magnitude. To show the data-range borders, dashed lines have been plotted above and below the solid trend-line. Of all nematode assemblages analysed, 78% were found within this range. When it is assumed that Fig. 5 provides a summary overview of the relationship between classical and molecular nematological analyses, it is notable, that the slope of the linear log-log regression across all our samples analysed in both ways (solid trend) is allometrically undistinguishable from unity (the slope 0.927±0.192 SE overlaps 1±0 SE; *P*<10^−5^). Discrepancies between counts and qPCR in one fifth of our samples are either due to underestimation or to overestimation of the nematode biomasses. On one hand, lacking appropriate molecular assays are a caveat that explains underestimation. For a number of non-monophyletic taxa such as the Rhabditidae, in fact, no molecular assays could be designed. Members of this family (*cp*-1) can respond very quickly to both local environmental changes (*e.g*. eutrification) as to microbial pulses. If such a family would be abundant in a given sample, this would automatically result in a drop of the coverage. On the other hand, though unusual, averages of body-mass values at genus level can be very different within a single family. This phenomenon can be illustrated by the “Plectidae minus *Anaplectus*”. In this group, the fresh weight per individual (and – most likely – the individual DNA content) varies substantially between genera (compare *Plectus* with *Wilsonema*, the latter being on average more than 7 times smaller than *Plectus* [13]). In our paper, the calibration curves were produced at genus level, and the quantification at family level was based on a qualitative check for Plectidae genera present in a given set of samples.

## Discussion

Comparison of the abundances of eight bacterivorous taxa during the entire experiment resulted in a diverse picture: for two taxa no difference was detected between the habitats, whereas six differed (four taxa were consistently more abundant in the field and two were present in significantly higher densities in the forest). Lumping the estimated nematode abundances into the feeding guild ‘bacterivores’ masks the taxon- and site-specific differences. One of those differences is the high density of Prismatolaimidae in the acidic moder. A factor that often contributes to this asymmetric distribution is pH, as some *Prismatolaimus* species prefer acidic conditions [48] and our forest moder might constitute an optimal environment for acidophilic bacterivores. Another of those differences was the distinct distribution of *Anaplectus* (Plectidae, Anaplectinae): throughout the seasons, this genus occurred at relatively constant density in the field, whereas it was virtually absent in the forest. This result confirms the outcome of a qualitative study of nematode communities in moder and mull (no stratification in humus-containing layer, organic matter and well-mixed mineral soil, pH 6.2–6.8) beech forest soils, where, *Anaplectus granulosus* was shown to be exclusively present in mull soils [49].

Regarding seasonal changes in bacterivorous nematodes, a decreasing trend was observed for Teratocephalidae and Plectidae in June and July (week 13 to 17 in Fig. 2), a period characterized by intense rainfall. Although precipitation data was recorded at a nearby weather station and not on the site itself, it would be tempting to attribute their absences to their movement to deeper soil layers. For several plant parasitic species, it has been shown that simulating intense rainfall hardly results in leaching of nematodes [50], [51]. As this ability to withstand leaching is unlikely to be specific for plant parasites alone, we see the absence of Teratocephalidae and Plectidae not as the (passive) result of any leaching below the sampling depth. However, members of these families could *actively* migrate downwards because of the leaching of certain groups of bacteria, their main resource. The passive transport of bacterial cells as a result of rainfall is a well-documented phenomenon [52], [53].

Intra-feeding guild heterogeneity is further illustrated by the fungivorous nematodes. The family Diphtherophoridae harbors two genera, *Diphtherophora* and *Tylolaimophorus*. Whereas representatives of the genus *Diphtherophora* were constantly present in the field from week 15 onwards (though in low densities), they were lacking in the adjacent forest. *Tylolaimophorus,* on the other hand, was the dominant fungivorous nematode in the forest (albeit in fluctuating densities). If soil acidity is so important for belowground Operational Taxonomic Units (OTUs) as suggested by Mulder *et al*. [45] and Wu *et al*. [54], than the difference in soil pH of our sites might (co)explain the observed patterns: in general, *Tylolaimophorus* spp. is known to be acidophilic [55], and its high abundance in the beech forest can be attributed to the acidic moder (pH = 3.7). The genus encompasses thirteen species [55], and we hypothesize that other, non-acidophilic *Tylolaimophorus* species might occur in the abandoned field (pH = 5.7).

A more pronounced, reverse response is observed for *Diphtherophora* (Fig. 3). Either the low pH of the forest soil was directly inhibiting the occurrence of *Diphtherophora* spp., or it is negatively affecting a part of the fungal community that provides an essential food resource for these nematodes. Members of other fungivorous families (Aphelenchidae and Aphelenchoididae) were respectively non-detectable or present in low amounts (late Autumn) in the acidic moder (Fig. 3B), whereas they occurred in the field at densities up to one individual per ml of soil, especially in the second half of the season. Hence, we hypothesize that (1) fungivorous nematodes are not as polyphagous as suggested [56] and that (2) Aphelenchidae and Aphelenchoididae feed on a part of the fungal community different from the segments used as a resource by *Tylolaimophorus.*

Regarding functional changes in nematodes, applied soil ecologists tend to use indices based on so-called guilds, a trait assemblage of nematode taxa sharing the same feeding habits and inferred function in the soil food web [14]. The current study did not aim to investigate effects of changes in the nutritional status of a soil food web, nor any other kind of environmental disturbance. Rather it shows that it is possible to monitor communities based on molecular methodology and in this way reveal numerous changes within feeding guilds that might give us more insight in the ecological functioning of soil biota.

In the last decades there have been several extensive studies on both spatial (*e.g.* [57], [58]) and temporal (*e.g.* [59], [60]) variation within nematode communities. Yeates *et al.* [59] monitored nematodes communities on 23 occasions over seven years under an annual and a perennial crop (3–4 sampling occasions per year). Their findings underlined the necessity of long-term monitoring (at least three years) to observe the effects of agricultural practices, and did not pay attention to seasonal fluctuations. Sohlenius and Boström [60] showed with a study of two annual time series (sampling monthly and every other month) and one long-term time series (10 sampling events over 25 years) in a Swedish pine forest soil, that variations within groups of nematode taxa of the same feeding type were larger compared to variations of the feeding groups as a whole. We did not observe major seasonal fluctuation in total numbers of nematodes as they did [60], but our data are in line with their conclusion stating that precipitation is one of the major drivers of changes in the nematode community composition.

So far, a number of practical obstacles like the time required for microscopic analysis, the limited number of informative morphological characters for some taxa, and the scarcity of people that can analyse nematode assemblages has restricted the number of intensive monitoring studies. DNA-based community analysis can lift such obstacles, and facilitate a wider use of nematodes as indicator for the biological soil quality. In essence, four different molecular approaches are currently used: direct sequencing, PCR DGGE, PCR T-RFLP and real time PCR [61].

Direct sequencing shares with PCR DGGE its qualitative rather than quantitative properties. In a careful comparison between microscopic and DGGE-based community analysis, Okada and Oba [25] found a reasonable match between the two methods. T-RFLP is a semi-quantitative PCR-based technique as well, and for the analysis of nematodes communities, the generation of a molecular framework is required. Recently, Donn *et al*. [26] reported on the effects of tillage on nematode communities, and as a start, a database with 516 partial SSU rDNA sequences from the sites under investigation was generated. The requirement of such a location-specific database and its semi-quantitative nature currently makes T-RFLP not an attractive method for routine analyses of nematodes.

Real time PCR is designed for quantification, and Jones *et al.* [62] were among the first to use this method for nematode community analysis. A local and small (74 SSU rDNA sequences) framework was made, and in a next step, community analysis procedure was tested based on the combined use of microscopy (for pre-selection) followed by real time PCR. The molecular procedure presented here allows for the analysis of nematode communities without any microscopic pre-selection because it is based on a considerably broader (2,400 taxa) full length SSU rDNA database that covers all major terrestrial and freshwater nematode taxa. It is noticed that marine nematode are greatly underrepresented in our framework, and consequently it can not be used for marine nematode assemblages yet. Still, a basic advantage of the detection framework illustrated here is its simplicity, as it only requires standard laboratory equipment.

In most soil nematological studies, data are presented at family or feeding guild level. To allow for a straightforward connection between the large body of ecological data on terrestrial nematode communities and the currently present molecular detection framework, it was decided to preferably develop assays at family level. Families often harbor multiple genera, and regression lines for family-specific primer combinations are based on one or more calibration curves produced on genus-level (*e.g.*, Fig. 4D). In case the relationship between real time PCR output (C_t_ value) is similar for the constituting genera, the ratio between these genera does not affect the accuracy of the results. However, some families such as Cephalobidae include genera with considerable body-size differences [13], [63] and, most likely, DNA contents. Therefore, only those genera that were present in the microscopic samples were included in the computation of the regression. As a consequence, the *R* ^2^ for Cephalobidae was slightly lower in comparison to most other primer combinations (Table 2). Hence, the accuracy of density levels of individual families is variable and depends on the variation in average DNA contents of the constituting genera.

On one hand, DNA-based research spans an enormous array of ecological disciplines and we believe that this study demonstrates – among others by showing for two adjacent, undisturbed areas that even 30 years after ending the agricultural practices the soil nematofauna barely seem to converge to the same assemblage – the ecological suitability of a quantitative PCR-based method for nematological and environmental purposes. On the other hand, our results also aim to contribute to the increase of the current knowledge of this phylum, given that the taxonomy of nematodes is still far from complete [64]. It has been shown that the analysis of datasets at genus level can provide more information when comparing analyses at family or order level. Using this DNA barcode-based tool, we have the possibility to work towards a complete view on time trends and soil patterns, enabling the nematode community to become unravelled.

## Supporting Information

Table S1 **Nematode densities (average ± standard error) in numbers of individuals per 100 ml elutriated soil at different times in a former arable field and its adjacent pristine beech forest.** Feeding guilds are given in capitals: B: bacterivore, F: fungivore, FP: facultative plant parasite, O: omnivore, P: predator. The weeks are defined as number of weeks after March 17.
(DOC)
