# Supplementary material for: SSU Ribosomal DNA-Based Monitoring of Nematode Assemblages Reveals Distinct Seasonal Fluctuations within Evolutionary Heterogeneous Feeding Guilds
Source: PLoS One. 2012 Oct 24;7(10):e47555. doi: 10.1371/journal.pone.0047555 (PMC3480422; doi:10.1371/journal.pone.0047555)
Supplement: Table S1 — Nematode densities (average ± standard error) in numbers of individuals per 100 ml elutriated soil at different times in a former arable field and its adjacent pristine beech forest. Feeding guilds are given in capitals: B: bacterivore, F: fungivore, FP: facultative plant parasite, O: omnivore, P: predator. The weeks are defined as number of weeks after March 17. (DOC) [file pone.0047555.s001.doc]

**Supplementary Table S1.** Nematode densities (average ± standard error) in numbers of individuals per 100 ml elutriated soil at different times in a former arable field and its adjacent pristine beech forest. Feeding guilds are given in capitals: B: bacterivore, F: fungivore, FP: facultative plant parasite, O: omnivore, P: predator. The weeks are defined as number of weeks after March 17.

| **i** | **Teratocephalidae** | | **Prismatolaimidae** | | **Plectidae** | | **Cephalobidae** | | ***Anaplectus*** | | **Alaimidae** | |
| --- | --- | --- | --- | --- | --- | --- | --- | --- | --- | --- | --- | --- |
|  | **B** | | **B** | | **B** | | **B** | | **B** | | **B** | |
| **Wk** | **‘Field’** | **‘Forest’** | **‘Field’** | **‘Forest’** | **‘Field’** | **‘Forest’** | **‘Field’** | **‘Forest’** | **‘Field’** | **‘Forest’** | **‘Field’** | **‘Forest’** |
| 0 | 190±121 | 144±79 | 13±7 | 2245±1159 | 100±27 | 33±5 | 328±79 | 166±62 | 40±16 | 0±0 | 0±0 | 0±0 |
| 2 | 15±12 | 12±12 | 12±7 | 733±74 | 27±11 | 2±2 | 248±100 | 4±0 | 8±5 | 0±0 | 24±23 | 0±0 |
| 4 | 52±29 | 9±8 | 5±4 | 1031±302 | 55±31 | 3±1 | 232±35 | 39±35 | 37±11 | 0±0 | 22±19 | 0±0 |
| 7 | 5±5 | 45±33 | 3±2 | 1833±1833 | 44±41 | 13±12 | 222±172 | 51±30 | 12±10 | 0±0 | 0±0 | 0±0 |
| 9 | 35±30 | 13±6 | 11±6 | 2949±2598 | 55±54 | 0±0 | 139±49 | 93±82 | 49±22 | 0±0 | 0±0 | 0±0 |
| 13 | 0±0 | 0±0 | 17±16 | 881±311 | 0±0 | 63±63 | 5±1 | 83±67 | 21±19 | 0±0 | 0±0 | 0±0 |
| 15 | 0±0 | 0±0 | 128±98 | 494±375 | 0±0 | 0±0 | 889±610 | 6±5 | 261±141 | 0±0 | 0±0 | 0±0 |
| 17 | 0±0 | 0±0 | 2±1 | 710±169 | 0±0 | 0±0 | 157±35 | 5±1 | 40±25 | 0±0 | 0±0 | 0±0 |
| 19 | 36±13 | 1±0 | 4±2 | 1107±1107 | 41±22 | 0±0 | 62±33 | 10±8 | 22±22 | 0±0 | 0±0 | 0±0 |
| 21 | 148±83 | 270±267 | 96±28 | 2735±601 | 645±412 | 86±85 | 306±148 | 2±2 | 935±730 | 0±0 | 53±17 | 38±38 |
| 23 | 84±79 | 0±0 | 10±8 | 669±669 | 91±59 | 0±0 | 337±220 | 0±0 | 308±188 | 0±0 | 35±24 | 0±0 |
| 25 | 145±50 | 652±340 | 13±5 | 3039±1907 | 294±98 | 18±14 | 338±162 | 5±3 | 69±23 | 0±0 | 27±9 | 0±0 |
| 27 | 12±12 | 0±0 | 28±16 | 33±32 | 180±87 | 0±0 | 271±99 | 2±2 | 18±11 | 0±0 | 106±52 | 0±0 |
| 29 | 76±76 | 404±209 | 20±7 | 4±4 | 354±157 | 10±6 | 189±85 | 14±2 | 154±120 | 0±0 | 27±13 | 0±0 |
| 33 | 62±41 | 550±223 | 87±53 | 542±73 | 662±370 | 163±66 | 370±126 | 125±63 | 218±185 | 5±5 | 83±60 | 50±29 |
| 35 | 978±459 | 568±204 | 130±76 | 2077±861 | 942±270 | 298±118 | 524±157 | 94±30 | 38±24 | 0±0 | 92±45 | 23±23 |
| 37 | 552±459 | 115 | 12±8 | 845 | 506±263 | 96 | 279±122 | 34 | 42±16 | 0 | 219±103 | 0 |
| 39 | 252±180 | 496±195 | 126±53 | 1217±1123 | 1714±870 | 68±31 | 1298±342 | 53±36 | 203±126 | 0±0 | 316±166 | 9±7 |

| **ii** | **Metateratocephalidae** | | **Monhysteridae** | | **Aphelenchoididae** | | **Aphelenchidae** | | ***Tylolaimophorus*** | | ***Diphtherophora*** | |
| --- | --- | --- | --- | --- | --- | --- | --- | --- | --- | --- | --- | --- |
|  | **B** | | **B** | | **F/FP** | | **F/FP** | | **F** | | **F** | |
| **Wk** | **‘Field’** | **‘Forest’** | **‘Field’** | **‘Forest’** | **‘Field’** | **‘Forest’** | **‘Field’** | **‘Forest’** | **‘Field’** | **‘Forest’** | **‘Field’** | **‘Forest’** |
| 0 | 0±0 | 550±410 | 393±94 | 468±185 | 0±0 | 0±0 | 4±4 | 0±0 | 8±8 | 101±87 | 1±1 | 0±0 |
| 2 | 0±0 | 0±0 | 156±36 | 52±28 | 0±0 | 0±0 | 4±3 | 0±0 | 81±78 | 82±82 | 1±1 | 0±0 |
| 4 | 67±67 | 94±11 | 414±160 | 108±108 | 0±0 | 0±0 | 11±4 | 0±0 | 8±7 | 19±18 | 1±0 | 0±0 |
| 7 | 0±0 | 591±591 | 133±125 | 112±37 | 0±0 | 0±0 | 2±2 | 0±0 | 0±0 | 5±2 | 9±6 | 0±0 |
| 9 | 0±0 | 1121 | 436±125 | 2900±2677 | 0±0 | 0±0 | 0±0 | 0±0 | 40±40 | 12±6 | 26±15 | 0±0 |
| 13 | 32±32 | 115±83 | 51±49 | 0±0 | 0±0 | 0±0 | 0±0 | 0±0 | 0±0 | 721±669 | 0±0 | 0±0 |
| 15 | 0±0 | 8±8 | 2139±1736 | 60±40 | 0±0 | 0±0 | 18±18 | 0±0 | 189±189 | 250±7 | 8±6 | 0±0 |
| 17 | 226±226 | 423±26 | 32±14 | 218±218 | 13±13 | 0±0 | 0±0 | 0±0 | 0±0 | 590±554 | 6±6 | 0±0 |
| 19 | 0±0 | 587±384 | 62±41 | 289±99 | 0±0 | 0±0 | 4±4 | 0±0 | 3±3 | 119±87 | 2±2 | 0±0 |
| 21 | 7±7 | 322±321 | 724±400 | 1196±172 | 0±0 | 0±0 | 70±25 | 0±0 | 4±4 | 157±149 | 36±21 | 0±0 |
| 23 | 0±0 | 3±3 | 279±88 | 409±409 | 0±0 | 0±0 | 30±4 | 0±0 | 22±21 | 21±2 | 19±5 | 0±0 |
| 25 | 47±47 | 82±69 | 221±64 | 459±169 | 2±2 | 0±0 | 36±10 | 0±0 | 0±0 | 114±13 | 43±30 | 0±0 |
| 27 | 30±30 | 1±1 | 18±11 | 0±0 | 2±2 | 0±0 | 45±16 | 0±0 | 15±11 | 8±8 | 36±21 | 0±0 |
| 29 | 68±68 | 182±85 | 69±12 | 0±0 | 2±1 | 0±0 | 39±23 | 0±0 | 261±150 | 1007±598 | 23±13 | 0±0 |
| 33 | 295±295 | 145±78 | 174±162 | 164±59 | 35±35 | 14±9 | 45±13 | 0±0 | 16±9 | 74±16 | 11±5 | 0±0 |
| 35 | 0±0 | 134±56 | 114±38 | 580±521 | 90±41 | 1±1 | 71±26 | 0±0 | 22±15 | 11±3 | 30±13 | 0±0 |
| 37 | 0±0 | 22 | 362±128 | 384 | 3±2 | 0 | 29±8 | 0 | 5±3 | 70 | 29±20 | 0 |
| 39 | 267±166 | 189±35 | 1114±445 | 128±128 | 35±19 | 8±6 | 55±15 | 1±1 | 2±2 | 472±61 | 10±5 | 0±0 |

| **iii** | **Dorylaimidae** | | **Mononchidae** | | **Mylonchulidae** | |
| --- | --- | --- | --- | --- | --- | --- |
|  | **O** | | **P** | | **P** | |
| **Wk** | **‘Field’** | **‘Forest’** | **‘Field’** | **‘Forest’** | **‘Field’** | **‘Forest’** |
| 0 | 1±1 | 6±5 | 0±0 | 0±0 | 0±0 | 0±0 |
| 2 | 6±6 | 0±0 | 0±0 | 0±0 | 0±0 | 0±0 |
| 4 | 3±2 | 8±8 | 0±0 | 0±0 | 0±0 | 0±0 |
| 7 | 0±0 | 6±6 | 16±12 | 0±0 | 0±0 | 0±0 |
| 9 | 0±0 | 4±3 | 9±7 | 2±2 | 2±1 | 4±4 |
| 13 | 0±0 | 0±0 | 4±2 | 0±0 | 1±1 | 1±1 |
| 15 | 0±0 | 0±0 | 3±3 | 0±0 | 1±1 | 0±0 |
| 17 | 10±10 | 0±0 | 17±12 | 0±0 | 1±0 | 9±3 |
| 19 | 108±85 | 0±0 | 2±1 | 0±0 | 0±0 | 1±1 |
| 21 | 349±141 | 182±182 | 22±14 | 0±0 | 0±0 | 0±0 |
| 23 | 43±43 | 5±5 | 43±37 | 3±3 | 0±0 | 0±0 |
| 25 | 74±74 | 37±28 | 48±44 | 0±0 | 3±3 | 0±0 |
| 27 | 8±8 | 60±60 | 1±1 | 0±0 | 0±0 | 0±0 |
| 29 | 71±71 | 48±2 | 5±3 | 4±4 | 0±0 | 0±0 |
| 33 | 28±28 | 66±39 | 8±8 | 2±2 | 0±0 | 0±0 |
| 35 | 379±41 | 396±107 | 5±1 | 5±3 | 0±0 | 0±0 |
| 37 | 93±57 | 83 | 8±8 | 3 | 0±0 | 0 |
| 39 | 189±114 | 125±16 | 6±2 | 1±0 | 0±0 | 0±0 |

From:

**SSU ribosomal DNA-based monitoring of nematode assemblages reveals distinct seasonal fluctuations within evolutionary heterogeneous feeding guilds**

Mariëtte T.W. Vervoort^1^, J. Arie Vonk^2^, Paul J.W. Mooijman^1^, Sven J.J. Van den Elsen^1^, Hanny H.B. Van Megen^1^, Peter Veenhuizen^3^, Renske Landeweert^3^, Jaap Bakker^1^, Christian Mulder^2^, and Johannes Helder^1^.

***^1^*** *Laboratory of Nematology, Department of Plant Sciences, Wageningen University (WUR), Box 9101, 6700 HB Wageningen, The Netherlands.*

***^2^*** *Laboratory for Ecological Risk Assessment, National Institute for Public Health and the Environment (RIVM), Box 1, 3720 BA Bilthoven, The Netherlands.*

***^3^*** *Laboratory for Soil and Crop Research (BLGG AgroXpertus), Box 170, 6700 AD Wageningen, The Netherlands.*

**Correspondence:**

Christian Mulder, Fax: ++31 30 2744413; E-mail: [Christian.Mulder@rivm.nl](mailto:Christian.Mulder@rivm.nl)

Johannes Helder, Fax: ++ 31 317 484254; E-mail: [Hans.Helder@wur.nl](mailto:Hans.Helder@wur.nl)
